# A PTEN-COL17A1 fusion gene and its novel regulatory role in Collagen XVII expression and GBM malignance

**DOI:** 10.18632/oncotarget.20526

**Published:** 2017-08-24

**Authors:** Xiaoyan Yan, Chuanbao Zhang, Tingyu Liang, Fan Yang, Haoyuan Wang, Fan Wu, Wen Wang, Zheng Wang, Wen Cheng, Jiangnan Xu, Tao Jiang, Jing Chen, Yaozhong Ding

**Affiliations:** ^1^ Department of Immunology, School of Basic Medical Sciences, Capital Medical University, Beijing 100069, China; ^2^ Beijing Neurosurgical Institute, Capital Medical University, Beijing 100050, China; ^3^ Department of Neurosurgery, Zhujiang Hospital, Southern Medical University, Guangzhou 510000, China; ^4^ Department of Neurosurgery, Beijing Tiantan Hospital, Capital Medical University, Beijing 100050, China; ^5^ The First Hospital of Baoding, Baoding, Hebei 071000, China

**Keywords:** Collagen XVII, COL17A1, gliomas, PTEN-COL17A1, fusion

## Abstract

Collagen XVII expression has recently been demonstrated to be correlated with the tumor malignance. While Collagen XVII is known to be widely distributed in neurons of the human brain, its precise role in pathogenesis of glioblastoma multiforme (GBM) is unknown. In this study, we identified and characterized a new PTEN-COL17A1 fusion gene in GMB using transcriptome sequencing. Although fusion gene did not result in measurable fusion protein production, its presence is accompanied with high levels of COL17A1 expression, revealed a novel regulatory mechanism of Collagen XVII expression by PTEN-COL17A1 gene fusion. Knocked down Collagen XVII expression in glioma cell lines resulted in decreased tumor invasiveness, along with significant reduction of MMP9 expression, while increased Collagen XVII expression promotes invasive activities of glioma cells and associated with GBM recurrences. Together, our results uncovered a new PTEN-COL17A1 fusion gene and its novel regulatory role in Collagen XVII expression and GBM malignance, and demonstrated that COL17A1 could serve as a useful prognostic biomarker and therapeutic targets for GBM.

## INTRODUCTION

Glioblastoma (GBM) is the most common primary brain tumor and among the most lethal forms of human cancer [1, 2]. Recent studies demonstrated that collagen gene expression and structural alterations are involved in GMB pathogenesis [3–5], Collagen XVII expression is upregulated in glioblastomas and promotes tumor cell adhesion [4], and the architecture of collagen in GBM are associated with patient survival [5].

Collagen XVII, also referred to as HemidesmosomesFraction4 (HD4), is a bullous pemphigoid antigen of 180-kDa (BP180) and a major structural hemidesmosomal transmembrane protein [6]. It exists in two forms: the full-length protein comprises of a homotrimeric transmembrane molecule of three180-kDa type XVII collagen A1 chains, and a soluble ectodomain of three 120-kDa polypeptides shed from the cell surface by furin-mediated proteolytic processing [7]. The NC16a domain of Collagen XVII contains the immunodominant epitopes of the bullous pemphigoid [8]. Previous studies have demonstrated that the shedding of the Collagen XVII ectodomain also acts as an antigen against the bullous pemphigoid [7, 9]. Junctional epidermolysis bullosa of late onset (JEB-lo) is an autosomal recessive disorder reflecting mutations in COL17A1 [10]. COL17A1 mutations have been associated with dominantly inherited epithelial recurrent erosion dystrophy (ERED) [11], aberrant Collagen XVII expression is implicated in the early atypia/malignant transformation of keratinocytes. Stelkovics et al showed that Collagen XVII was significantly elevated in squamous cell carcinoma (SCC) at invasive tumor fronts, comprised of tumor adjacent stroma, endothelium, and histiocytes, compared to keratinocytes of normal skin and benign lesions [12]. Collagen XVII was exclusively detected in malignant melanocytic tumors and melanoma cell lines, and antibodies targeting the 507–529 aa region of Collagen XVII could promote apoptosis and inhibit proliferation in a melanoma cell line (HT199) [13]. Collagen XVII expression increased with higher TNM stages, decreasing the disease-free and poor outcomes in colorectal carcinoma, and Collagen XVII overexpression promoted invasion in murine colon carcinoma cells [14].

In the nervous system, Collagen XVII has been found to be often co-localized with its epithelial ligand BPAG1 and complexing with various laminins, in Muller glial cells, photoreceptors of bovine and rat tissues, while localize predominantly to the soma and proximal axons of human neurons [15], its precise role in pathogenesis of glioblastoma multiforme (GBM) is unknown.

Chromosomal translocations generate fusion genes and produce oncogenic abnormal fusion proteins. Many fusion genes have been identified and studied in hematological malignancies and solid tumors [7, 9]. Transcriptome sequencing is a powerful tool for detecting gene fusions in tumors. Increasing numbers of recurrent fusion genes have been identified using next-generation sequencing technologies [16]. Singh et al first reported the FGFR3-TACC3 fusion transcript in glioblastoma multiforme (GBM) cases using high-throughput transcriptome sequencing [17]. The FGFR3-TACC3 fusion protein induces constitutive kinase activation and mitotic and chromosomal segregation defects. Veronique et al reported the fusion of EGFR with several partners in GBM using large-scale whole genome and transcriptome sequencing. EGFR-SEPT14, the most frequent recurrent fusion transcript, activates STAT3 signaling [18].

We recently examined the fusion landscape of WHO-grade II-IV glioma patients using RNA sequencing analysis, 214 fusion transcripts in 272 glioma patients were identified, and revealed a novel PTPRZ1-MET fusion transcript in secondary GBMs (sGBMs) [19]. Here, we further identified and characterized a new PTEN-COL17A1 fusion gene in human gliomas, our results demonstrated that Collagen XVII promoted cell invasion through MMP9-mediated hydrolysis of the cell matrix, and revealed a novel regulatory role of PTEN-COL17A1 fusion in Collagen XVII expression and GBM malignance.

## RESULTS

### RNA-Sequencing analysis identified PTEN-COL17A1 fusion gene occurrences

**Figure 1:**
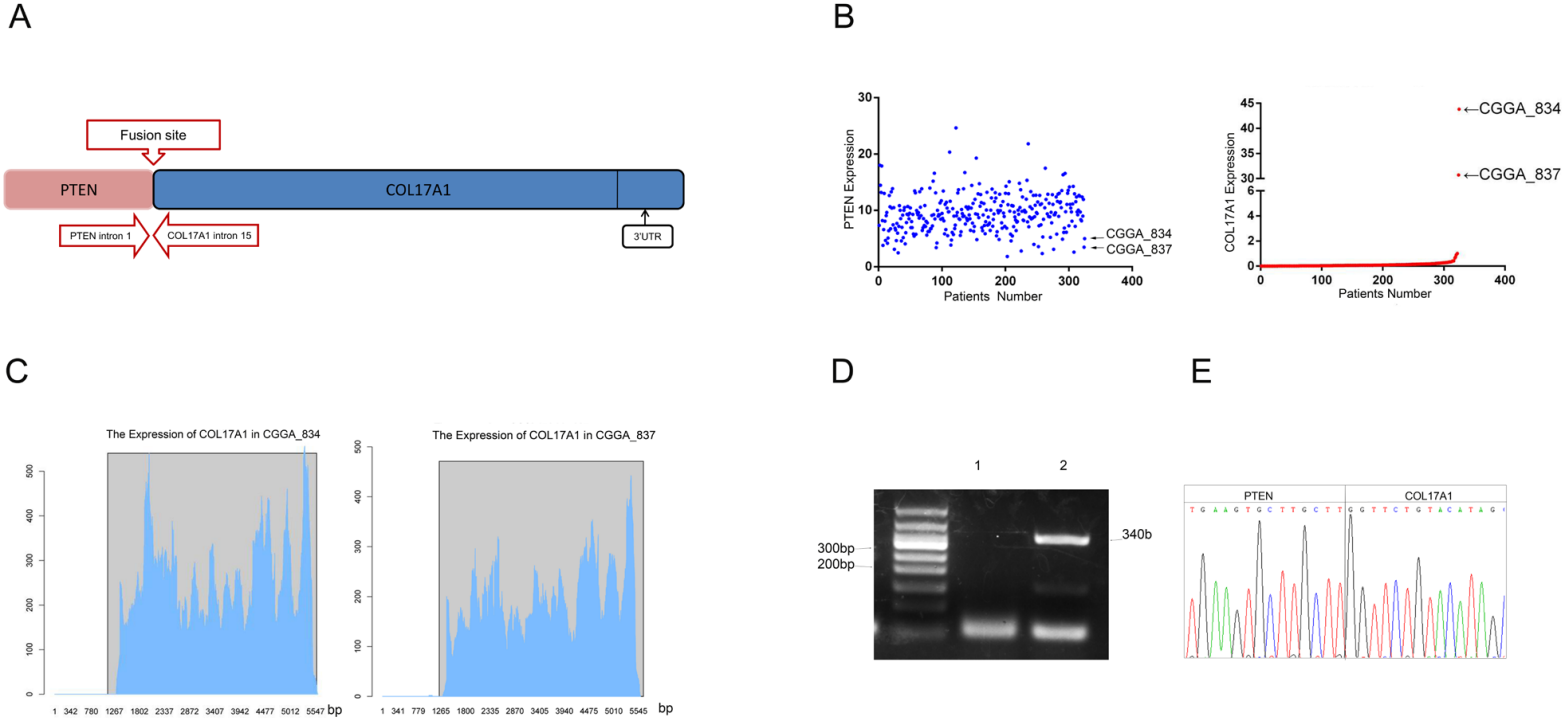
Identification and characterization of a novel PTEN-COL17A1 fusion in gliomas using RNA sequencing **(A)** Schematic of PTEN-COL17A1 fusion derived from transcriptome sequencing. Genomic fusion of PTEN intron 1 with intron 14 of COL17A1, The solid black arrows indicate the 3’UTR of the fusion. **(B)** The expression of PTEN and COL17A1 in 325 WHO-grade II-IV glioma patients using RNA sequencing analysis. **(C)** The expression of COL17A1 in two PTEN-COL17A1 fusion gene-positive samples was analyzed by sequencing. RNA expression increased approximately after 1265 bases of COL17A1 in CGGA-834 and CGGA-837. **(D)** RT-PCR validation of PTEN-COL17A1. Lane 1, Negative control; Lane 2, CGGA837; Lane M, 50-bp DNA ladder. **(E)** Sanger sequencing result showing the nucleotide sequence at the breakpoint of PTEN-COL17A1 fusion in CGGA-837.

Our previous study revealed 214 fusion transcripts in 272 WHO-grade II-IV glioma patients, including 67 in-frame fusions and147 out-of-frame fusions. We further found that Reads of the PTEN-COL17A1fusion gene site were positive in two cases (CGGA-834 and CGGA-837). The clinical characteristics of CGGA-834 and CGGA-837 patients showed in Table 2. PTEN was located in the plus strand of chromosome ten, and COL17A1 was located in the antisense strand of the same chromosome. The precise breakpoints in intron 1 of PTEN and intron 14 of COL17A1 are illustrated in (Figure 1A). We measured the gene expression levels of both genes, and only COL17A1 revealed an increase in mRNA expression (Figure 1B). RNA expression increased after the fifteenth exon of COL17A1 in both CGGA-834 and CGGA-837 (Figure 1C). These results suggested that the biological role of this fusion likely act through increasing expression of COL17A1, rather than PTEN. RT-PCR for PTEN-COL17A1 was performed to further validate the existence of this fusion gene. A pair of primers crossing the fusion site, located at intron 1 of PTEN and intron 14 of COL17A1, was designed. PCR amplification and Sanger sequencing crossing the breakpoint of CGGA_837 samples were applied (Figure 1D). The results confirmed the existence of the fusion event in the sample, consistent with the results of the RNA-Seq analysis. Unfortunately, due to the lack of a sufficient amount of tissue, we were not able to validated fusion gene in sample CGGA_834.

**Table 1:** The sequence of siRNA used in this study

Primer	Sequence
Si-COL17A1-1	Sense5’-ACGAGAUGGAACUGAAGUCdTdT-3’ Antisense 5’-GACUUCAGUUCCAUCUCGUdTdT-3’
Si-COL17A1-2	Sense 5’-GUCACUGAGAGAAUUGUCAdTdT-3’ Antisense 5’-UGACAAUUCUCUCAGUGACdTdT-3’
Si-COL17A1-3	Sense 5’-CCACAAGACUUACAUCCUUdTdT-3’ Antisense 5’-AAGGAUGUAAGUCUUGUGGdTdT-3’
Negative control	Sense5’-UUCUCCGAACGUGUCACGUTT-3’ Antisense 5’-ACGUGACACGUUCGGAGAATT-3’

**Table 2:** Clinical characteristics of CGGA-834 and CGGA-837 patients

	CGGA-834	CGGA-837
Age-year	25	57
Sex	Male	Male
WHO grade	II	IV
TCGA subtypes	Classical	Classical
Histology	Oligoastrocytoma (OA)	Glioblastoma (GBM)
EGFRvIII	no	no
IDH1-R132	no	no
IDH2-R172	no	no
IDH	no	no
MGMT	yes	yes
OS	1151	432

EGFR: epidermal growth factor receptor; MGMT: methylguanine DNA-methyltransferase; OS: Overall survival.

### Prediction and detection of the putative protein encoded by the PTEN-COL17A1 fusion gene

**Figure 2:**
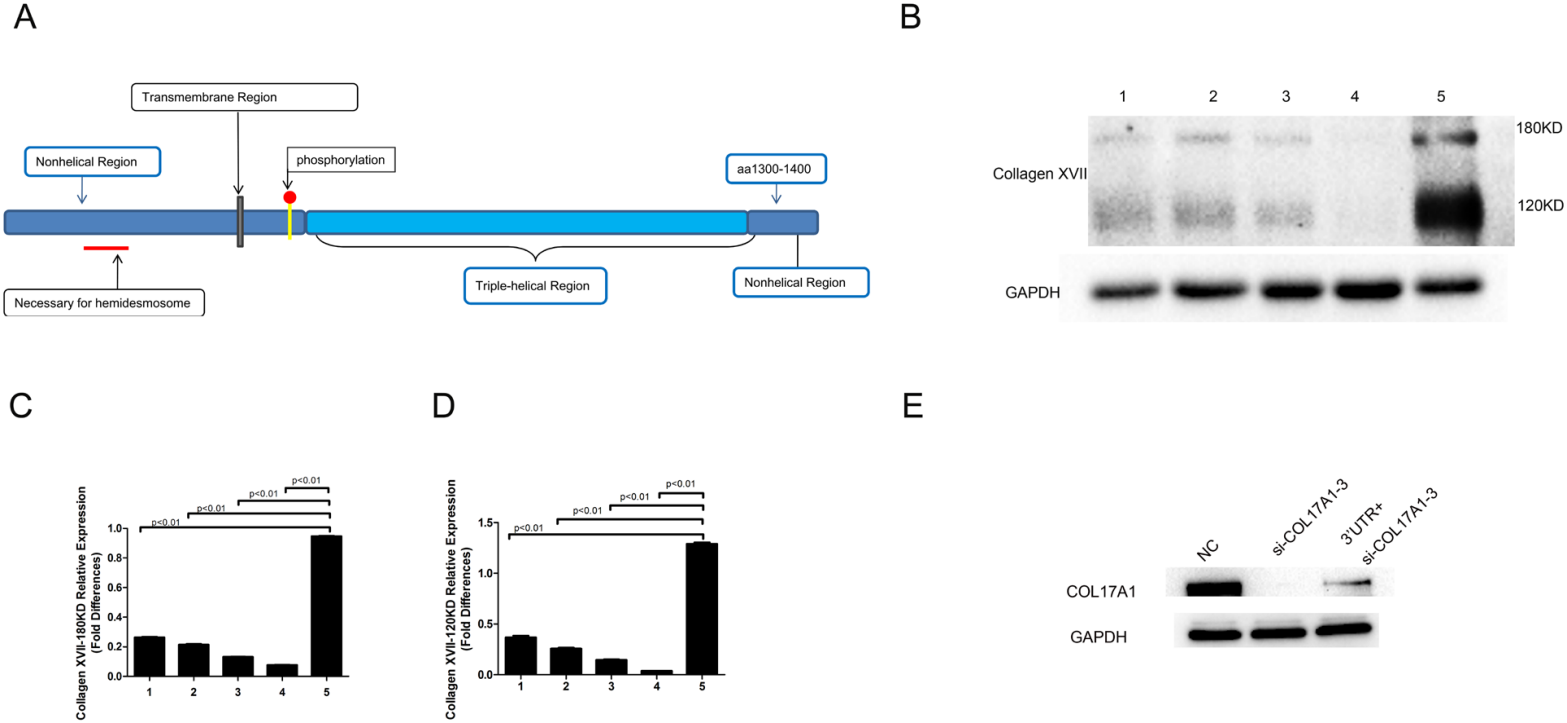
Increased level of Collagen XVII expression was observed in the PTEN-COL17A1 gene fusion positive samples **(A)** Schematic of Collagen XVII monoclonal antibodies against the amino acid region from the 1300th to 1400th amino acid. **(B)** Collagen XVII expression was determined by western blotting. Normal human brain tissue (1.2.3) PTEN-COL17A1-negative samples (4) and the PTEN-COL17A1-positive sample CGGA837(5). 180KDa and 120KDa proteins of COL17A1 expression were significantly increased in CGGA-837compared to the controls. **(C-D)** 180KDa and 120KDa proteins of COL17A1weresignificantly increased as measured by densitometry (normalized to GAPDH) (p<0.01). **(E)** 3'UTR of PTEN-COL17A1 fusion reduced the efficiency of siRNA (si-COL17A1-3) knock down for Collagen XVII expression (p<0.01).

The SIB ExPASy Bioinformatics Resources Portal was used to analyze and predict the putative protein encoded by the PTEN-COL17A1 fusion gene. According to the translation mechanism, the PTEN-COL17A1fusion gene sequence could encode a protein of 982 amino acids with a molecular weight of 108 kDa. To determine whether the PTEN-COL17A1 fusion gene produce a protein of 108 kDa, we used western blot analysis to examine three normal controls, one negative control and CGGA-837. We selected monoclonal antibodies against the sequence located in the region from the 1300th to 1400th amino acid of Collagen XVII (Figure 2A), which detects the proteins encoded by wild-type COL17A1 and the PTEN-COL17A1 fusion gene. The protein encoded by the PTEN-COL17A1 fusion gene was 108 kDa, and the proteins encoded by wild-type Collagen XVII were 180 and 120 kDa. The different molecular weights would distinguish these proteins. As shown in Figure 2B, the 108-kDa band was not detectable in CGGA-837, indicating the fusion gene did not produce measurable full-length fusion protein. But importantly, Collagen XVII expression was significantly increased in CGGA-837 compared to the controls (P<0.01) (Figures 2C-2D), indicating a positive regulatory role of the PTEN-COL17A1 fusion gene in Collagen XVII expression. As RNA sequencing analysis revealed that the expression of the 3'UTR of the PTEN-COL17A1 fusion significantly increased (Figure 1C). The 3'UTR of the PTEN-COL17A1 fusion and COL17A1have the same sequences. Considering that miRNAs play a role in the fine regulation of gene expression through the 3'UTR [20], It is possible that the 3 'UTR region of the PTEN-COL17A1 fusion gene could absorb microRNAs targeting COL17A1, thus up-regulate endogenous COL17A1 expression and Collagen XVII production. To test this possibility, we showed that the high levels 3'UTR of PTEN-COL17A1 fusion indeed reduced the efficiency of siRNA knock down for COL17A1 expression (Figure 2E), supporting a mechanism that the 3 'UTR region of the PTEN-COL17A1 fusion gene could interfere the negative regulatory machinery of COL17A1 gene regulation, thus up-regulate endogenous COL17A1 expression and Collagen XVII production.

### COL17A1 knockdown decreases invasiveness but did not affect migration in glioma cells

**Figure 3:**
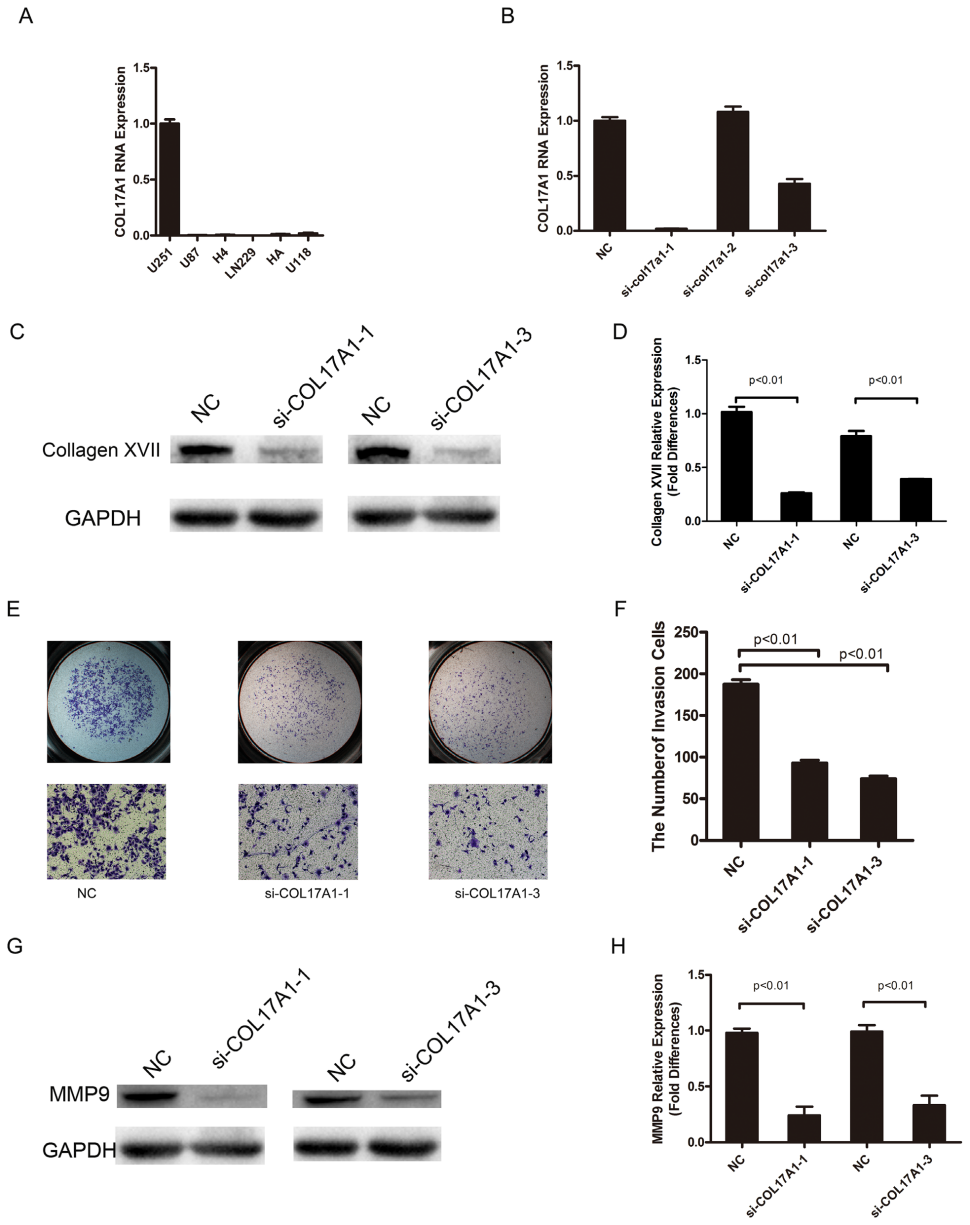
COL17A1 knockdown decreases invasiveness **(A)** Real-time PCR analysis of COL17A1 expression in 6 glioma cell lines. **(B)** Real-time PCR analysis of COL17A1 expression after the application of siRNA in U251 glioma cell lines. **(C-D)** After transduction, Collagen XVII expression was determined by western blotting. The expression of Collagen XVII was significantly reduced by siRNA targeting COL17A1 as measured by densitometry (normalized to GAPDH) (p<0.01). **(E-F)** The number of invaded cells significantly decreased in COL17A1 knockdown cells compared with the control. The expression of MMP9 was significantly reduced by siRNA targeting COL17A1, as measured using western blotting **(G)** and measured by densitometry **(H)** (normalized to GAPDH) (p<0.01).

Real-time PCR was performed on five glioma cell lines, including U251, U87, H4, LN229, and U118, and the normal human astrocyte cell line HA. All cell lines showed COL17A1 expression. U251 showed the highest expression of COL17A1 (Figure 3A), and this cell line was chosen for further studies. The U251 cell lines were treated with COL17A1 siRNA, and COL17A1 knockdown was observed following 48 hours after siRNA transfection. Quantitative real-time PCR and western blot analysis showed that si-COL17A1-1 and si-COL17A1-3 exhibited the most effective knockdown effects (P<0.01) (Figures 3B-3C).

We next performed matrigel and migration assays to examine how Collagen XVII expression affects the invasion and migration of glioma cells. The invasiveness of U251 cells decreased following COL17A1 reduction compared to cells transfected with non-targeting siRNA (P<0.01) (Figure 3A), and MMP9 expression was significantly reduced after knocking down COL17A1 (P<0.01) (Figure 3E), while COL17A1 knockdown had little effects on the rate of migration in U251 glioma cell lines.

### Increased Collagen XVII expression promotes invasive activities of glioma cells and associated with GBM recurrences

**Figure 4:**
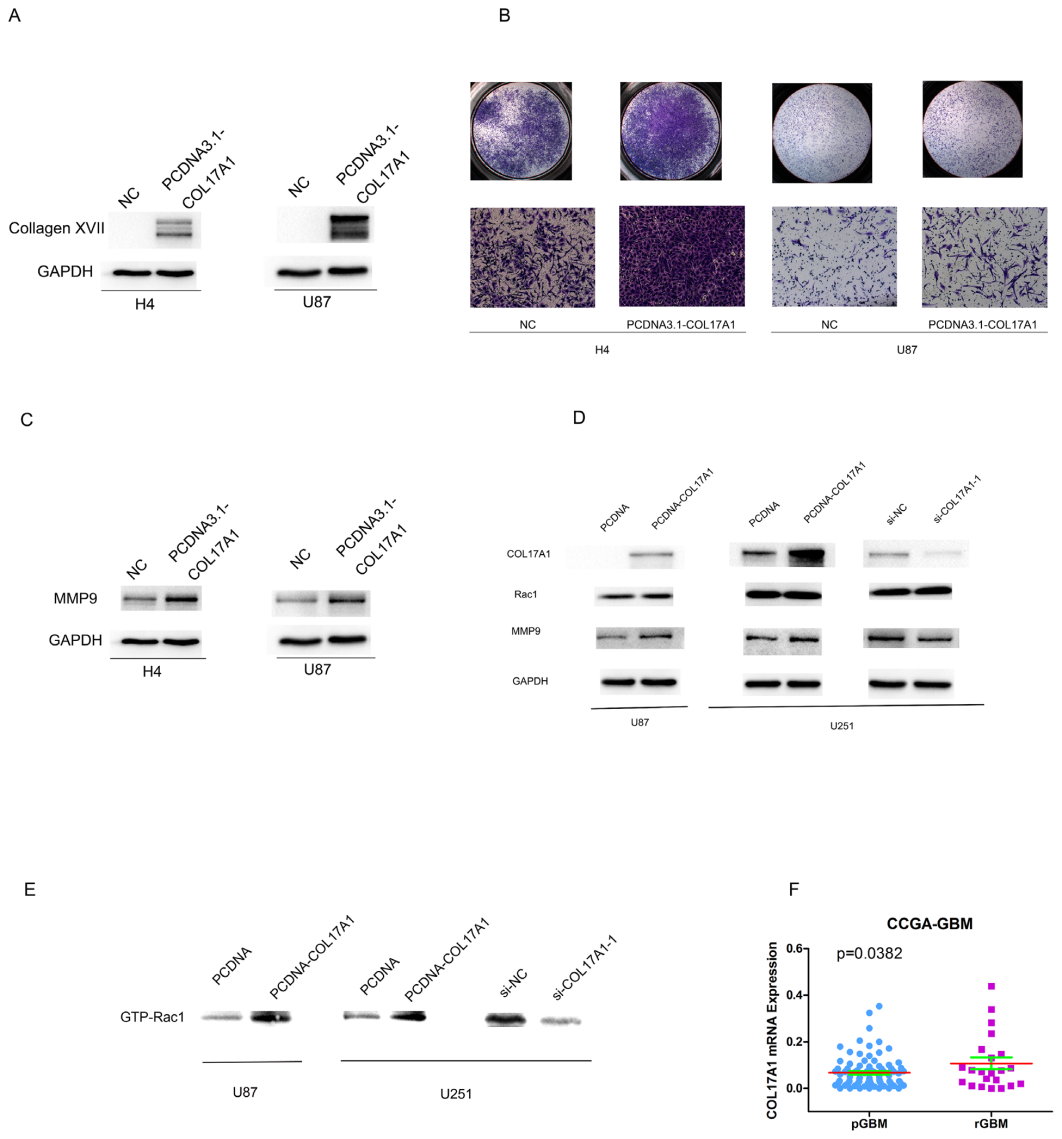
Increased Collagen XVII expression promotes invasive activities of glioma cells and associated with GBM recurrences **(A)** After Col17A1 gene transfection, Collagen XVII expression was determined by Western blotting **(B)** The number of invaded cells significantly increased in Collagen XVII over-expressing cells compared with the control(p<0.01). **(C)** The expression of MMP9 was significantly increased in COL17A1 over expressing cells measured by western blotting (p<0.01). **(D)** The expression of Rac showed no obvious change between Collagen XVII over-expressing cells and control **(E)** COL17A1 expression directly affects activated form of Rac expression (GTP-Rac1) (p<0.01). **(F)** Collagen XVII expression levels were significantly higher in recurrent GBM (rGBM) patients than that in Glioblastoma multiforme (pGBM) patients (p<0.05).

We also performed COL17A1 gene transfection experiments to further determine the influence of Collagen XVII expression on their invasive abilities of glioma cell lines. Western blot analysis measured the protein expression of COL17A1 in H4 cells or U87 after transfected with pcDNA3.1- COL17A1 or pcDNA3.1-NC vectors (P<0.01) (Figure 4A). We employed cell invasion assays to determine effects of COL17A1 over-expression on malignant progression and metastasis. The results showed that numbers of migratory H4 and U87 cells which transfected with pcDNA3.1- COL17A1 vectors were significantly higher compared to the pcDNA3.1-NC groups after transfected for 48h (P<0.01) (Figure 4B), and the protein expression of MMP9 was significantly increased after expression of COL17A1 (P<0.01) (Figure 4C), (Figure 4D). Rac1 is a member of the Rho family of small GTPases, and a upstream regulator for MMP9 gene expression [21, 22]. We thus examined Rac and its activated form (GTP-Rac1) expression. As shown in Figures 4D, 4E, COL17A1 expression directly affects activated form of Rac expression, indicating that COL17A1 regulate MMP9 expression likely through GTP-Rac1 activity in glioma, Together these data demonstrated that Increased Collagen XVII expression promotes the invasive activities of glioma cells.

To further explore the biological relevance of COL17A1 expression in GMB pathogenesis, we analyzed gene expression data of 87 Primary Glioblastoma multiforme (pGBM) patients and 22 recurrent GBM (rGBM) patients obtained from CGGA. As summarized in Figure 4F, COL17A1 was upregulated in 22 recurrent GBM patients compared to 87 pGBM patients (P<0.05). These results further reinforced an important role of Collagen XVII in GBM malignance and recurrence.

## DISCUSSION

Chromosomal translocations leading to production of oncogenic fusion proteins, and play important roles in the pathogenesis of human cancer [3]. In current study, we further identified and characterized a new PTEN-COL17A1 fusion gene in human gliomas. GMB is known for its heterogeneity [4, 5, 15, 23], which enables this cancer with an ability to adapt to and evade the anti-tumor therapy. 3.1% of GBMs harbors oncogenic chromosomal translocations that fuse in-frame the tyrosine kinase coding domains of FGFR1 or FGFR3 to the transforming acidic coiled-coil (TACC) coding domains of TACC1 or TACC3 [17], here we reported less than 1% of GMB positive of PTEN-COL17A1gene fusion, further reflecting the diversity of GMB, demonstrating the importance of precise diagnosis and therapy for each individual patient. More importantly, we revealed a novel mechanism for fusion gene to regulate gene expression and tumor pathogenesis. Instead of acting through fusion protein production it can also could use other mechanism to influence expression of genes important in tumor pathogenesis, in this case, upregulate COL17A1 expression.

It has been found that COL17A1 is expressed in malignant but not in benign melanocytic tumors [13], recent reports also showed the expression of COL17A1 in colon epithelium and the association of increased COL17A1 expression with poor outcome in colorectal carcinoma [14]; suspension survival mediated by PP2A-STAT3-COLXVII determines tumor initiation and metastasis in cancer stem cells [24], demonstrating its important role in tumor pathogenesis. In this study, three glioma cell lines were transiently transfected with corresponding si-RNA or gene expression plasmid for COL17A1, we showed that increased or decreased COL17A1 expression resulted in increased or decreased tumor invasiveness, along with significant increment or reduction of MMP9 expression (Figures 3-4), demonstrating an important role of Collagen XVII in promoting cell invasion through the MMP9-hydrolyzed cell matrix, pointing to an important role of Collagen XVII in GMB malignance and recurrence.

RNA-Sequencing analysis showed that PTEN was not significantly changed in the samples (CGGA-834, CGGA-837) with positive PTEN-COL17A1 fusion gene expression. Although we were not able to detect full-length PTEN-COL17A fusion protein by Western blotting, RNA sequencing analysis revealed that the expression of the 3'UTR of the PTEN-COL17A1 fusion significantly increased (Figure 1C), and western blot analysis showed that COL17A1 protein expression significantly increased in CGGA-837 compared with controls. Thus, the biological role of this fusion is likely acting through increasing expression of COL17A1.

In summary, in the present study, RNA sequencing analysis revealed existences of the PTEN-COL17A1 fusion gene in GBM. COL17A1 expression was significantly increased in PTEN-COL17A1 fusion gene-positive samples, COL17A1 could promote cell invasion through the MMP9-hydrolyzed cell matrix, and increased Collagen XVII expression are associated with GBM recurrences. We also revealed a novel mechanism for fusion gene to regulate gene expression through increasing 3'UTR of expression, and demonstrated that COL17A1 could serve as a novel prognostic biomarker and therapeutic targets for GBM.

## MATERIALS AND METHODS

### Clinical glioma samples

Clinical specimen collection was approved by the institutional review board (IRB) at Tiantan Hospital in accordance with the principles expressed at the declaration of Helsinki. Consent was obtained from each patient prior to specimen collection. The data do not contain any information that might lead to the identification of the patients.

### Expression analysis of PTEN-COL17A1 fusion genes

The structure and quantitative mRNA expression of the PTEN-COL17A1 fusion were analyzed using the Illumina HiSeq2000 transcriptome sequencing data from the Chinese Glioma Genome Atlas (CGGA). These data can be partially retrieved from the Chinese Glioma Genome Atlas (CGGA) (http://www.cgga.org.cn).

### RNA extraction and cDNA synthesis

The specimens used in experiments included one CGGA-837 sample, in which the PTEN-COL17A1 fusion was positively detected by transcriptome sequencing; 7 GBM specimens; and 3 non-tumor brain tissue samples derived from non-tumor patients after the removal of brain tissue for decompression.

Total RNA was extracted from all frozen tissue samples and 6 cell lines using Trizol reagent (Invitrogen, Carlsbad, CA, USA) according to the manufacturer’s instructions. First-strand cDNA was synthesized from1000 ng of total RNA using Oligo (dT) 18 primer and RevertAid First Strand cDNA Synthesis Kit (Thermo, Waltham, CA, USA).

### Gene fusion validation

A forward primer targeting the PTEN gene prior to the fusion site (5'-ATTTCAGCGTATGTTGGTCTCTACAC -3') and a reverse primer targeting the COL17A1 gene (5'- GAAGTCAAGGTGGACAACAATCATATT-3') were designed to detect the fusion transcripts. PCR was performed using the following procedure: 95°C for 2 min, followed by 35 cycles of 95°C for 30 sec, 59°C for 30sec and 72°C for 30 sec, with a final step at 72°C for 5 min. The presence of the fusion gene in the CGGA-837 sample was confirmed using direct Sanger sequencing.

### Cell lines and cell culture

Six human glioma cell lines, including U251, U87, H4, LN229, and U118, and human astrocytes (HA) were obtained and cultured at 37°C and 5% CO2 in DMEM/F12 medium (Gibco, Carlsbad, CA, USA) supplemented with 10% fetal bovine serum (FBS, Gibco, Carlsbad, CA, USA).

### Real-time quantitative RT-PCR

The cDNAs were amplified by qRT-PCR using SYBR Select Master Mix(Applied Biosystems, Carlsbad, CA, USA) on an ABI PRISM 7000 Sequence Detection System (Applied Biosystems, Foster City, CA, USA) with the forward primer 5'- TTACCCGCCATGCGTATGAAG-3' and reverse primer: 5'- CAGTCGAACTCGAATTTCACTCT-3'. qRT-PCR was performed using the following procedure: an initial denaturation step at 95°C for 5 min, followed by 40 cycles of denaturation at 95°C for 15 sec and elongation at 60°C for 45 sec. The comparative 2-ΔΔCt method was used for relative quantification, and statistical analysis with glyceraldehyde-3-phosphate dehydrogenase (GAPDH) was used as the endogenous control.

### Western blot analysis

The cells were lysed using RIPA buffer (Cell Signaling Technology, Danvers, MA, USA), supernatants were collected, and the protein concentrations were measured using a BCA Protein Assay Kit (Thermo, Waltham, CA, USA). SDS-PAGE was performed, subsequently transferred to PVDF membranes (Merck Millipore, Darmstadt, Germany) and incubated with anti-COL17A1(Immunogen: Synthetic peptide within Human COL17A1 aa 1300-1400) (Abcam, Cambridge, MA, USA), anti-MMP9 (Abcam, Cambridge, MA, USA), and anti-GAPDH (CWbiotech, Beijing, China). Rac Activation Assay Kit (NewEast Biosciences, USA) was used to detect the active form of RAC1 by pull-down assay. The blots were developed using the Bio-Rad GelDoc XR System and analyzed using Image Lab 5.0 (Bio-Rad Laboratories, Hercules, CA, USA).

### RNA interference and transfection

COL17A1 siRNA 1-3 were designed according to the siRNA design principles. The siRNA molecules were synthesized at GenePharma in China. COL17A1 knockdown was achieved following transient transfection using three different human fascin siRNA duplexes. In all experiments, we used a non-targeting oligonucleotide to exclude the nonspecific off-target effects of RNA interference (Table 1). siRNA Transfection Reagent was used for all transfections (Polyplus-transfection Inc, New York, NY, USA). The expression vector (pcDNA3.1) that express COL17A1 (pcDNA3.1- COL17A1) and a negative control (pcDNA3.1-NC) were transfected into H4 cells or U87 cells using X-tremeGENE HP DNA Transfection Reagent (Roche, Mannheim Germany) according to the manufacturer’s instructions. The cells were cultured 24 h prior to transfection, and cells transfected with pcDNA3.1- COL17A1 and pcDNA3.1-NC were harvested after 48 h. The expression vector (pcDNA3.1) that express 3’UTR (pcDNA3.1-3’UTR) and a negative control (pcDNA3.1-NC) were transfected into U251 cells or U87 cells using X-tremeGENE HP DNA Transfection Reagent (Roche, Mannheim Germany) according to the manufacturer’s instructions.

### Invasion and migration assays

An admixture of 12μl of Matrigel (Corning Incorporated Life Sciences, Tewksbury, MA, USA) and 48 μl of DMEM/F12 medium was added to transwell (Corning Incorporated Life Sciences, Tewksbury, MA, USA). The lower chamber of the transwell was filled with the DMEM/F12 medium supplemented with 10% FBS. The cells were incubated at 37°C for 24 h in with 5% CO_2_ and subsequently stained with crystal violet. The migrated cells that migrated into the lower surface were counted. Cells in five random fields of view at 100 × magnification were scored as the average number per field of view.

### Statistical analysis

The gene expression data of 87 Primary Glioblastoma multiforme (pGBM) patients and 22 recurrent GBM (rGBM) patients obtained from CGGA. The GraphPad Prism 5.0 statistical software was used to assess the statistical significance between pGBM and rGBM. Student’s two-tailed t test was used to determine significant differences. All data are presented as the mean ± standard error. The value was considered statistically significant at p<0.05.
